# Needle stick injury and associated factors among acupuncture practitioners: a cross-sectional study in China

**DOI:** 10.3389/fpubh.2024.1515889

**Published:** 2024-12-18

**Authors:** Wenjing Jiang, Ying Liu, Li Cao, Ping Zhou, Anna Dai, Juan Tang

**Affiliations:** ^1^Department of Hospital Infection Management, Zigong First People’s Hospital, Zigong, China; ^2^Department of Nursing, Sichuan Vocational College of Health and Rehabilitation, Zigong, China; ^3^Department of Infectious Diseases, Zigong First People’s Hospital, Zigong, China

**Keywords:** needle stick injury, risk factors, acupuncture, healthcare workers, China

## Abstract

**Background:**

Needle stick injury (NSI) is one of the most common and severe occupational hazards for healthcare workers (HCWs), leading to both physical harm and psychological distress and ultimately affecting patient safety. Previous studies on NSI were predominantly focused on general clinical practice, and limited research has targeted specifical NSI occurring in acupuncture practice in China, which has the greatest use of acupuncture.

**Objective:**

This study aimed to investigate NSI and associated factors among acupuncture practitioners in China.

**Methods:**

A cross-sectional online survey was conducted among acupuncture practitioners across 98 hospitals in southwest China from April to May 2024. A researcher-developed questionnaire was used to collect participants’ experiences of NSI, general information, and knowledge, behavior, and risk perception related to occupational exposure. Multivariate logistic regression was employed to examine factors associated with NSI.

**Results:**

A total of 578 acupuncture practitioners completed the questionnaire, among whom 34.3% experienced at least one NSI in the past three years, yet 46.0% of these incidents were not reported. Factors associated with an increased risk of NSIs included postgraduate education or higher (OR = 2.174, 95% CI: 1.020, 4.634), high probability of occupational exposure (OR = 2.940, 95% CI: 1.826, 4.735), moderate perception of exposure severity (OR = 9.149, 95% CI: 1.948, 42.97), and high perception of exposure severity (OR = 7.025, 95% CI: 1.497, 32.969). Conversely, factors associated with a reduced risk of NSIs included females (OR = OR = 0.576, 95% CI: 0.379, 0.876), nurses (OR = 0.396, 95% CI: 0.210, 0.746), working in other professions (OR = 0.362, 95% CI: 0.151, 0.868), adherence to standard preventive measures (OR = 0.396, 95% CI: 0.210, 0.746), and consistent adherence to post-exposure procedures (OR = 0.092, 95% CI: 0.021, 0.398).

**Conclusion:**

NSI is common among acupuncture practitioners in Chinese medical institutions, and under-reporting is significant. Our findings suggest that standard prevention strategies, adherence to exposure protocols, enhanced training, and effective reporting policies may help reduce NSI and improve reporting rates.

## 1 Introduction

Worldwide, needle stick injury (NSI) is one of the most common and severe occupational hazards for healthcare workers (HCWs) (1). NSI refers to an injury caused by a needle or a sharp object (2). A systematic review and meta-analysis showed that the worldwide pooled prevalence of NSI among HCWs was 56.2% during career time and 32.4% in the past year (1). In the US, an estimated 385,000 NSIs occur annually among HCWs in hospital settings, with similar incidents reported in other healthcare settings (3). In developing countries, the incidence and prevalence of NSI are even higher due to a lack of policies, culture, guidelines, and training related to NSI management in these resource-limited regions (1). In China, a national survey revealed that the incidence of NSI among nurses following contact with blood and body fluids was as high as 52.1% (4). In Shanghai, 1.53% of HCWs reported experiencing at least one NSI in the past month, underscoring the high risk of NSI within the healthcare sector (5).

On the other hand, underreporting of NSI is also highly prevalent among HCWs due to multiple reasons, such as poor knowledge and awareness of NSI, concerns about privacy and losing a job, and busy working schedules (6). A systematic review and meta-analysis focusing on nursing students showed that the pooled prevalence of NSI was 35%, with the highest prevalence in Asia (39.7%). However, 63% of nursing students did not report NSI (2). The issue of underreporting is more threatening in developing countries, with an estimated half of NSI going unreported (7–9). One study showed that the reporting rate of NSI was only 64.07%, underscoring a critical underreporting problem and insufficient focus on occupational safety in this field (10). The high incidence of NSI, coupled with the low reporting rate, poses a significant challenge to the management and prevention of NSI (2, 6).

NSI remains the most common route of exposure to bloodborne pathogens and a leading cause of infectious disease among HCWs when the needle is contaminated with blood or other body fluids (11, 12). According to the World Health Organization (WHO) (13), NSI contributes to 39, 37, and 4.4% of hepatitis C virus (HCV), hepatitis B virus (HBV), and human immunodeficiency virus (HIV) infections, respectively. A study across 14 geographical regions found that NSI among HCWs led to approximately 66,000 HBV infections, 16,000 HCV infections, and 1,000 HIV cases each year (14). According to the United States Occupational Safety and Health Administration (OSHA), approximately 5.6 million HCWs in the US are at risk of occupational exposure to bloodborne pathogens due to NSI (15). In China, HCWs’ probabilities of exposure to HVB, HVC, and HIV due to NSI were 6–30%, B, 0–7%, and 0.2–0.5%, respectively (16).

Although extensive studies have been conducted to investigate NSI among various HCWs in various countries, most of them were focused on NSI during general clinical practice. Few studies have targeted specific NSIs occurring in acupuncture practice in China, a country with the greatest use of acupuncture and the highest rate of HBV infection (17). Acupuncture is a traditional Chinese therapeutic method and involves the insertion of fine needles into specific points on the body to regulate Qi and blood circulation, promote self-healing, and achieve health benefits (18, 19). Due to its well-demonstrated therapeutic effects, acupuncture has gained widespread application in clinical practice, playing an increasingly important role both in China and outside China (20–22). Acupuncture practitioners, due to the nature of their work, are particularly susceptible to NSI. NSI not only incurs physical harm but also causes severe psychological distress, such as anxiety, fear, stress, insomnia, and depression, which can negatively affect job satisfaction and quality of life (4, 23). Furthermore, these consequences may potentially compromise patient safety and increase healthcare delivery costs (24, 25). A study conducted at a tertiary hospital in China involving 1,585 acupuncture practitioners found that 164 workers experienced a total of 231 acupuncture needle exposures over three years, with an incidence rate of 10.35% (10). Therefore, ensuring the occupational safety of acupuncture practitioners is crucial.

Despite the significant risk, there is still a lack of research specifically addressing the prevalence and influencing factors of NSI among acupuncture practitioners in China. To fill this gap, our study investigated NSIs and associated factors among acupuncture practitioners in Southwest China. This research aimed to identify critical issues related to NSI, providing valuable insights for the future development of educational programs, training initiatives, and safety systems to protect acupuncture practitioners from occupational hazards.

## 2 Methods

### 2.1 Study design and participants

A cross-sectional online study was conducted at 98 hospitals of various levels (primary, secondary, and tertiary) across 21 cities in southwest China, covering a diverse range of acupuncture practitioners, from April to May 2024. A convenience sampling method was adopted to recruit all eligible participants who met the following criteria: (1) age ≥ 18, (2) having practiced acupuncture therapy for at least six months, (3) able to complete the questionnaire survey with normal mental and cognitive function, and (4) voluntary participation in this study. Participants with NSI that occurred beyond the study period (over three years prior to data collection) were excluded from the study.

The sample size was determined using a single population proportion formula: $n=\frac{u_{a/2}^{2}\pi \left(1-\pi \right)}{\delta^{2}}$ , which is commonly used in cross-sectional studies to calculate sample sizes (4). Here, a was 0.05, u was 1.96, π was 50%, and δ was 0.05, leading to a minimal sample size requirement of 384. Considering potential participant rejection and invalid questionnaires, we further expanded our sample size to a larger number. A total of 601 participants completed questionnaires, among whom 23 provided invalid questionnaires and thus were removed from the analysis, leading to a final sample of 578 (effective response rate: 96.17%). The study is reported according to the Strengthening the Reporting of Observational Studies in Epidemiology (STROBE) reporting guideline.

### 2.2 Study procedure

Ethical approval was obtained from the Institutional Review Board of Zigong First People’s Hospital (No. 2024093). Data were collected through Sojump, one of the largest and most popular online survey platforms in China (26). Prior to the survey, study flyers were sent to the directors of the Infection Prevention and Control Departments from 98 hospitals via WeChat, the most commonly used social media platform in China. The directors then arranged for the eligible acupuncture practitioners to complete the survey. Those who were interested in this study could scan the QR code on WeChat to complete the survey after providing electronic informed consent. Participants were informed that no personal identification information was collected and that they could withdraw at any time. To ensure data completeness, the survey could only be submitted after it was fully completed. To avoid duplicate questionnaires, each smartphone or computer ID could only be used to submit the survey once.

### 2.3 Measurements

A researcher-designed questionnaire based on the literature, policies, and guidelines related to occupational exposure was used as the primary survey instrument in this study (27, 28). The final version of the questionnaire was determined based on feedback from experts in this field. The questionnaire comprises four parts. The first part includes participants’ general information, such as gender, age, education level, working years, profession, professional title, and hospital level. It also incorporates questions related to hepatitis B vaccine and serological status. The second part consists of 16 questions on knowledge and behaviors related to occupational protection. This section starts with occupational protection training, including whether participants have received training, the last time of training, and the way of knowledge acquisition. Additionally, it includes questions regarding the definitions of occupational exposure and standard prophylaxis, transmission routes of bloodborne pathogens, and preventive measures.

The third section focuses on the risk assessment of occupational exposure, comprising four questions that examine participants’ perceptions of the probability, severity, and coping ability associated with such exposure. Finally, participants who have experienced NSI were also required to complete the fourth section, which comprises ten questions related to the epidemiological characteristics of these injuries. These include the frequency of the injury, the type of acupuncture needle causing the injury, the place of the injury, the procedure/task during which the injury occurred, etc.

### 2.4 Data analysis

All data were analyzed using IBM SPSS Statistics 24. Frequencies and percentages were used to describe categorical variables. Pearson chi-square tests were utilized to examine differences among various groups. A multivariate logistic regression analysis was performed to investigate the associations between sample characteristics and NSI and identify independent factors associated with NSI. The model was selected based on its ability to assess multiple independent variables simultaneously and to identify factors that may not be apparent in bivariate analysis. To enhance the model’s accuracy, the following steps were taken: (1) potential confounding variables were identified and included in the model, (2) a backward elimination procedure was employed to ensure that only significant predictors were included, and (3) multicollinearity was checked using variance inflation factors (VIFs) to ensure that predictors were not highly correlated. A 2-sided *p* value of less than 0.05 indicated statistical significance.

## 3 Results

### 3.1 Participant characteristics

The basic characteristics of the participants are presented in Table 1. Among the 578 participants included in the study, most were female (60.7%), aged between 25and 40 (66.4%), had undergraduate education or below (92.6%), and were formal employees (88.2%). About half of the participants were physicians (51.7%) and worked in tertiary hospitals (52.2%). Most participants had received the HBV vaccine (81.0%) and obtained occupational protection knowledge mainly from the hospital (61.4%). Half of them (53.5%) had been tested for HBV, HCV, TP, and HIV during the last year.

**Table 1:** Basic characteristics of the participants (*n* = 578).

Characteristics	Participants (*n*)	Percent (%)
Gender		
Male	227	39.3
Female	351	60.7
Age (years)		
<25	101	17.5
25–30	177	30.6
30–40	207	35.8
≥40	93	16.1
Education level		
Junior college or below	224	38.8
Undergraduate	311	53.8
Postgraduate or above	43	7.4
Work experience (years)		
≤1	96	16.6
1–5	121	20.9
5–10	165	28.5
>10	196	33.9
Profession		
Physician	299	51.7
Nurse	128	22.1
Technician	108	18.7
Other	43	7.4
Professional title		
No	107	18.5
Primary	259	44.8
Middle	166	28.7
Advanced	46	8
Hospital level		
Primary	105	18.2
Secondary	171	29.6
Tertiary	302	52.2
Employment type		
Temporary employee	68	11.8
Formal employee	510	88.2
Have you received the HBV vaccine?		
No	110	19.0
Yes	468	81.0
Have you been tested for HBV, HCV, TP, and HIV in the last year?		
No	269	46.5
Yes	309	53.5
Which is the most important way for you to obtain occupational protection knowledge?		
Hospital	355	61.4
Department	151	26.1
School	59	10.2
Other	13	2.2

HBV, hepatitis B virus; HCV, hepatitis C virus; TP, treponema pallidum; HIV, human immunodeficiency virus.

### 3.2 Characteristics of NSI

A total of 198 participants (34.3%) reported experiencing NSI at least once in the past three years. However, a significant proportion of 46.0% (195 out of 424) failed to report NSI to the hospital’s Infection Control Committee. Table 2 shows the detailed characteristics of the NSI cases. NSI most frequently occurred once (46.5%) or twice (23.7%) in the treatment room (41.3%) or the physiotherapy room (38.7%). NSI was mainly attributed to filiform needles (84.7%), with unknown pathogen sources (59.1%). Additionally, most participants did not wear gloves when the NSI occurred (75%), and 41.3% had NSI during needle removal. After the NSI, most participants received treatment according to the standard process (78.8). In addition, 32.4% had antibody tests, 20.3% had liver function tests, 29.0% reported experiencing anxiety, and 5.7% expressed significant fear.

**Table 2:** Characteristics of acupuncture NSI (*n* = 198).

Characteristics	Participants (*n*)	Percent (%)
Frequency of the NSI		
1	92	46.5 (92/198)
2	47	23.7 (47/198)
3	22	11.1 (22/198)
4	13	6.6 (13/198)
≥5	24	12.1 (24/198)
Type of acupuncture needle causing the NSI		
Filiform needle	359	84.7 (359/424)
Intradermal needle	29	6.8 (29/424)
Cutaneous needle	10	2.4 (10/424)
Three-edged needle	6	1.4 (6/424)
Small knife needle	6	1.4 (6/424)
Other	14	3.3 (14/424)
The place where the NSI occurred		
Treatment room	175	41.3 (175/424)
Physiotherapy room	164	38.7 (164/424)
Inpatient ward	57	13.4 (57/424)
Outpatient clinic	24	5.7 (24/424)
Other	4	0.9 (4/424)
Procedure/task during which the NSI occurred		
Preparation of materials	64	15.1 (64/424)
Insertion of needle	76	17.9 (76/424)
Removal of needle	175	41.3 (175/424)
Needle waste collection	87	20.5 (87/424)
Another person’s recklessness	14	3.3 (14/424)
Other	8	1.9 (8/424)
Whether wearing gloves when the NSI occurred		
No	318	75.0 (318/424)
Yes	106	25.0 (106/424)
Wound treatment after NSI		
Treatment according to the standard process	334	78.8 (334/424)
Treatment not following the standard process	65	15.3 (65/424)
No action	25	5.9 (25/424)
Report to the infection control committee		
Yes	229	54.0 (229/424)
No (do not know the reporting process)	56	13.2 (56/424)
No (consider it unnecessary)	139	32.8 (139/424)
Type of bloodborne pathogen exposure (multiple-choice question)		
HIV	66	10.5 (66/626)
HBV	83	13.3 (83/626)
HCV	50	8.0 (50/626)
TP	57	9.1 (57/626)
No/Unknown	370	59.1 (169/626)
Prevention measures after the NSI (multiple-choice question)		
Immunoglobulin injection	80	13.3 (80/601)
Antibody test	195	32.4 (195/601)
Liver function test	122	20.3 (122/601)
Vaccine injection	65	10.8 (65/601)
Drug therapy	94	15.6 (94/601)
Other	45	7.5 (45/601)
The main psychological reactions after the NSI		
Normal (after the proper post-exposure treatment)	189	44.6 (189/424)
No idea	11	2.6 (11/424)
Anxious	123	29.0 (123/424)
Fearful	24	5.7 (24/424)
Indifferent	77	18.2 (77/424)

NSI, needle stick injury; HBV, hepatitis B virus; HCV, hepatitis C virus; TP, treponema pallidum; HIV, human immunodeficiency virus.

### 3.3 Factors associated with NSI

We first compared the differences in all sample characteristics between participants with and without NSI using Pearson chi-square tests. The two groups showed statistically significant differences in various sample characteristics, such as gender, education level, profession, employment type, and training experience, which are listed in Table 3. These significant factors were further included in the multivariate logistic regression analysis, and the remaining significant factors are shown in Table 4. Factors associated with an increased risk of NSIs included postgraduate education or higher (OR = 2.174, 95% CI: 1.020, 4.634), high probability of occupational exposure (OR = 2.940, 95% CI: 1.826, 4.735), moderate perception of exposure severity (OR = 9.149, 95% CI: 1.948, 42.97), and high perception of exposure severity (OR = 7.025, 95% CI: 1.497, 32.969). In contrast, factors associated with a reduced risk of NSIs included: females (OR = 0.576, 95% CI: 0.379, 0.876), nurses (OR = 0.396, 95% CI: 0.210, 0.746), working in other professions (OR = 0.362, 95% CI: 0.151, 0.868), adherence to standard preventive measures (OR = 0.396, 95% CI: 0.210, 0.746), and consistent adherence to post-exposure procedures (OR = 0.092, 95% CI: 0.021, 0.398).

**Table 3:** Univariate analysis for acupuncture NSI.

Variables	NSI n (%)		*χ^2^*	*P*
	No	Yes		
Gender				
Male	127	100(44.1)	15.930	<0.001
Female	253	98(27.9)		
Education level				
Junior college or below	152	72(32.1)	14.169	0.001
Undergraduate	211	100(32.2)		
Postgraduate or above	17	26(60.5)		
Profession				
Physician	170	129(43.1)	32.691	<0.001
Nurse	108	20(15.6)		
Technician	69	39(36.1)		
Other	33	10(23.3)		
Employment type				
Temporary employee	37	31(45.6)	4.394	0.036
Formal employee	343	167(32.7)		
Have you taken the training course on occupational exposure?				
No	9	13(59.1)	6.263	0.012
Yes	371	185(33.3)		
When was the last time you received occupational protection training?				
Within 3 months	232	99(29.9)	12.057	0.007
Within 4–6 months	51	35(40.7)		
Within 7–12 months	43	17(28.3)		
>1 year, none, or no idea	54	47(46.5)		
Do you follow standard prevention strategies during work?				
No / Unknown	202	132(39.5)	9.738	0.002
Yes	178	66(27.0)		
What do you think the probability level of occupational exposure at your workplace is?				
Low	171	49(22.3)	32.300	<0.001
General	112	56(33.3)		
High	97	93(48.9)		
What do you think is the severity level of occupational exposure at your workplace?				
Low	31	13(29.5)	8.202	0.017
General	29	30(50.8)		
High	320	155(32.6)		
Can you follow standard procedures after occupational exposure?				
Disagree	23	17(42.5)	24.413	<0.001
General	5	19(79.2)		
Agree	352	162(31.5)		
Do you think that following the standard procedures after occupational exposure will not cause infection?				
Disagree	71	51(41.8)	11.479	0.003
General	48	39(44.8)		
Agree	261	108(29.3)		

NSI, needle stick injury.

**Table 4:** Multivariate analysis of acupuncture NSI.

Variables	*B*	OR	OR (95%CI)	*P*
Gender				
Male	Ref	1		
Female	−0.551	0.576	(0.379, 0.876)	0.010
Education level				
Junior college or below	Ref	1		
Undergraduate	−0.224	0.800	(0.521, 1.227)	0.306
Postgraduate or above	0.777	2.174	(1.020, 4.634)	0.044
Profession				
Physician	Ref	1		
Nurse	−0.928	0.396	(0.210, 0.746)	0.004
Technician	−0.125	0.883	(0.537, 1.451)	0.623
Other	−1.017	0.362	(0.151, 0.868)	0.023
Do you follow standard prevention strategies during work?				
No / Unknown	Ref	1		
Yes	−0.928	0.396	(0.210, 0.746)	0.004
What do you think the probability level of occupational exposure at your workplace is?				
Low	Ref	1		
General	0.365	1.440	(0.877, 2.364)	0.150
High	1.079	2.940	(1.826, 4.735)	0.000
What do you think is the severity level of occupational exposure at your workplace?				
Low	Ref	1		
General	2.214	9.149	(1.948, 42.97)	0.005
High	1.949	7.025	(1.497, 32.969)	0.013
Can you follow standard procedures after occupational exposure?				
Disagree	Ref	1		
General	−0.603	0.547	(0.093, 3.239)	0.507
Agree	−2.385	0.092	(0.021, 0.398)	0.001

NSI, needle stick injury.

## 4 Discussion

To our knowledge, this multicenter cross-sectional study is the first to specifically address the issues of NSIs among acupuncture practitioners in hospitals across southwest China. Our findings revealed that approximately one-third of acupuncture practitioners experienced at least one NSI in the past three years. Yet, nearly half of them never reported these incidents, indicating a double challenge of high prevalence and low reporting. Multiple factors have been identified to be associated with NSI, such as gender, education level, profession, adherence to preventive measures, perceived likelihood and severity of exposure, and compliance with post-exposure protocols. These results highlight the need for effective prevention and management strategies to reduce and eliminate NSI.

### 4.1 Prevalence of NSI among acupuncture practitioners

Our results indicate that acupuncture practitioners are at a significantly high risk for NSI, with an incidence rate of 34.3%, similar to the reported 32.86% in a previous national survey of HCWs in China (29). However, this rate was higher than the 10.35% reported in a study conducted at a single tertiary Traditional Chinese Medicine hospital (10). Additionally, 12.1% of acupuncture practitioners in our study reported more than five NSIs over the past three years, compared to 10.35% reported in a previous study (10). This difference is likely due to our study’s retrospective review of NSIs over the past three years and a broader research scope involving multiple centers and hospitals at varying levels. Our study also revealed a high under-reporting rate for NSI at 46.0%, consistent with previous studies reporting rates of 35–50% (7–9). Several studies have identified common reasons for not reporting, including the prolonged reporting process, concerns over losing a job, and underestimating the hazards of NSI (7, 29, 30). The consistently high under-reporting rates across various studies underscore the urgent need to establish standard and comprehensive reporting systems, both in China and globally. Such systems are crucial for ensuring accurate data collection and improving the safety of HCWs worldwide.

### 4.2 Characteristics of NSI among acupuncture practitioners

In this study, we found that filiform needles accounted for most NSI exposure cases. This is likely due to the widespread use of filiform needles in acupuncture treatment and the handling difficulties due to small size. Our study also found that most injuries occurred in treatment and physiotherapy rooms during the process of withdrawing acupuncture needles, which was consistent with previous findings (10). Therefore, enhancing protective measures in these high-risk areas and developing specialized sharp containers for filiform needles could be crucial preventive measures (31, 32). NSI has been shown to transmit over 20 bloodborne pathogens, including HBV, HCV, HIV, and syphilis, contributing to most occupational infections (33). In our study, 40.9% of participants were exposed to these pathogens, with HBV being the primary source (13.3%), which was congruent with previous findings (5, 23, 34). Additionally, we found that 10.5% of the needles linked to NSI were contaminated with HIV, a rate significantly higher than the 3–6% reported in earlier studies (23, 34). Notably, our study revealed that a considerable proportion of acupuncture practitioners experienced anxiety (29.0%) and fear (5.7%) following NSI, similar to the anxiety rates of 25–30% reported in previous studies (4, 23). For example, a study by Li et al. showed that 12.6% of HCWs who experienced NSI considered quitting, 3.5% wanted to change jobs, and 1.8% reported that the stress affected their families (23). Thus, NSI not only poses a risk to acupuncture practitioners’ physical health but also causes significant psychological stress.

### 4.3 Factors associated with an increased risk of NSIs among acupuncture practitioners

Based on our findings, several factors were associated with an increased risk of NSIs, including holding a postgraduate or higher degree, being a physician, having a high probability of occupational exposure, and having a moderate to high perception of occupational exposure severity. Practitioners with postgraduate or higher degrees were found to have a significantly higher risk of NSIs, consistent with previous research (7). This increased risk may be due to challenges in translating extensive theoretical knowledge into practical acupuncture skills. Although highly educated practitioners possess strong theoretical understanding, they may lack sufficient hands-on experience, leading to less precise needle handling and higher risk of injury. Moreover, the current medical education system often prioritizes theoretical knowledge and advanced techniques over practical training in safety protocols and needle stick injury (NSI) prevention. As a result, even highly educated practitioners may not receive sufficient training on the practical aspects of NSI prevention, leaving them vulnerable to occupational risks. However, evidence supports that education and training focused on NSI prevention and infection control are effective measures (4, 29, 35). Furthermore, the risk of NSI was higher among doctors than nurses and other medical staff, likely due to doctors’ more frequent use of acupuncture needles (10). Similarly, a higher probability of occupation exposure risk indicates higher chances of contacting dangerous objects, leading to a higher risk of NSI. Additionally, moderate and high perceptions of occupational exposure severity were associated with a higher risk of NSI. It is noteworthy that an excessively high perception of occupational exposure severity may represent an over-exaggeration and anxiety of the occupation hazards, which may paradoxically impede occupational protective practices, thereby augmenting the risk of exposure. Conversely, risk perception of occupational exposure at a low level may represent a normal level of awareness of potential hazards and psychological response, which can keep individuals vigilant while not over-anxious, thereby reducing the risk of NSI (36, 37).

### 4.4 Factors associated with a reduced risk of NSIs among acupuncture practitioners

Our findings shown that female acupuncture practitioners were associated with a reduced risk of NSI. This may be due to factors such as greater adherence to safety protocols, more cautious handling of needles, and higher awareness of occupational risks, which collectively contribute to a reduced likelihood of injury. Additionally, our study demonstrated that adherence to standard prevention strategies in the workplace was linked to a reduced risk of NSI, consistent with previous research findings (8, 9). Standard precautions are widely recognized as the most effective approach for preventing and controlling nosocomial infections, protecting HCWs and patients, and ensuring public safety (38). Previous studies have shown that using double gloves can significantly reduce exposure to bloodborne pathogens on sharp instruments (8, 35, 39). However, while these findings are promising, additional research is needed to evaluate the long-term effectiveness and practicality of these preventive strategies in real-world settings. Additionally, our study further found that adhering to standard procedures after exposure was associated with a lower risk of NSI. Therefore, it is imperative that we not only focus on pre-exposure prevention measures but also emphasize the importance of standardized post-exposure management.

## 5 Study limitations

The study has several limitations that should be acknowledged. First, the cross-sectional design limits our ability to establish causal relationships between NSI and associated factors; future longitudinal studies are needed to confirm these relationships. Second, the use of a convenience sampling method may have introduced selection bias regarding study settings and participants. Future studies may consider using random sampling to get a more representative sample. Third, information on NSI experience was collected based on self-report, which is subject to recall bias. Additionally, as this was a self-administered questionnaire, the lack of probing questions may have led to incomplete or superficial responses, potentially underestimating or overlooking certain aspects of NSI experiences. While we do not anticipate that this limitation would substantially alter the overall trends observed, future studies could benefit from combining self-report methods with interviewer-administered surveys or other objective assessments to enhance data accuracy and depth. Lastly, since the data were collected from southwest China, the regional specificity may limit the generalizability of the findings to other areas. Studies in other parts of China are needed to validate our findings further.

## 6 Conclusion

This study shows that NSI is common among acupuncture practitioners in Chinese medical institutions, yet under-reporting of such exposures remains prevalent. Multiple factors have been identified to be associated with the risk of NSI, such as gender, education level, profession, adherence to standard preventive measures, probability of occupational exposure, severity of occupational exposure, and compliance with post-exposure procedures. To reduce exposure and improve reporting, it is crucial to implement standard prevention strategies, ensure adherence to exposure prevention protocols, provide comprehensive education and training on exposure prevention and control, and establish precise exposure reporting policies. We recommend that managers focus on the occupational safety of acupuncture practitioners and introduce targeted interventions to enhance their protection.

## Data Availability

The raw data supporting the conclusions of this article will be made available by the authors, without undue reservation.
